# Brain Derived Neurotrophic Factor (*BDNF*) Expression Is Regulated by MicroRNAs miR-26a and miR-26b Allele-Specific Binding

**DOI:** 10.1371/journal.pone.0028656

**Published:** 2011-12-14

**Authors:** Viviana Caputo, Lorenzo Sinibaldi, Alessia Fiorentino, Chiara Parisi, Caterina Catalanotto, Augusto Pasini, Carlo Cogoni, Antonio Pizzuti

**Affiliations:** 1 Department of Experimental Medicine, Sapienza University of Rome, Rome, Italy; 2 European Brain Research Institute, Rome, Italy; 3 Mendel Laboratory, IRCCS “Casa Sollievo della Sofferenza”, San Giovanni Rotondo, Italy; 4 Department of Molecular Medicine, Sapienza University of Rome, Rome, Italy; 5 Department of Cellular Biotechnology and Hematology, Sapienza University of Rome, Rome, Italy; 6 Division of Child Neuropsychiatry, Department of Neuroscience, University of Rome Tor Vergata, Rome, Italy; Florida State University, United States of America

## Abstract

Brain-derived neurotrophic factor (BDNF) is a neurotrophin that plays an essential role in neuronal development and plasticity. MicroRNA (miRNAs) are small non-coding RNAs of about 22-nucleotides in length regulating gene expression at post-transcriptional level. In this study we explore the role of miRNAs as post-transcriptional inhibitors of *BDNF* and the effect of 3′UTR sequence variations on miRNAs binding capacity. Using an *in silico* approach we identified a group of miRNAs putatively regulating *BDNF* expression and binding to *BDNF* 3′UTR polymorphic sequences. Luciferase assays demonstrated that these miRNAs (miR-26a1/2 and miR-26b) downregulates *BDNF* expression and that the presence of the variant alleles of two single nucleotide polymorphisms (rs11030100 and rs11030099) mapping in *BDNF* 3′UTR specifically abrogates miRNAs targeting. Furthermore we found a high linkage disequilibrium rate between rs11030100, rs11030099 and the non-synonymous coding variant rs6265 (Val66Met), which modulates *BDNF* mRNA localization and protein intracellular trafficking. Such observation led to hypothesize that miR-26s mediated regulation could extend to rs6265 leading to an allelic imbalance with potentially functional effects, such as peptide's localization and activity-dependent secretion. Since rs6265 has been previously implicated in various neuropsychiatric disorders, we evaluated the distribution of rs11030100, rs11030099 and rs6265 both in a control and schizophrenic group, but no significant difference in allele frequencies emerged. In conclusion, in the present study we identified two novel miRNAs regulating *BDNF* expression and the first *BDNF* 3′UTR functional variants altering miRNAs-*BDNF* binding.

## Introduction

Brain-derived neurotrophic factor (BDNF) is a member of the neurotrophins family, which consists of small secreted proteins playing crucial roles in activity-dependent processes, such as synapses development and plasticity [1]. BDNF promotes neuronal survival and differentiation of specific populations of embryonic neurons in peripheral and central nervous system and shows also in adulthood a crucial regulatory role in key functions, including neuronal homeostasis and brain plasticity-related processes such as learning and memory [2]–[3].

Several DNA variants mapping within the *BDNF* genomic region have been associated with a number of human traits, such as performance on intelligence tests, various cognitive functions, personality, and memory [4]–[8]. Notably, there are many evidences for *BDNF* contribution to the pathogenesis of several neuropsychiatric disorders. To date *BDNF* has been reported to be associated with schizophrenia [9]–[12], Parkinson's disease [13]–[16], addictive substance use or dependence [17], Alzheimer's disease [18]–[19], bipolar disorder or depression [20]–[23] and obsessive-compulsive disorder [24]. In particular, the common non-conservative single nucleotide polymorphism (SNP) rs6265 (G>A), resulting in a Valine to Methionine aminoacid change at codon 66 in the pro-domain of BDNF protein (pro-BDNF), has been extensively analysed in several neuropsychiatric disorder through linkage and association studies leading to conflicting results [25]–[29]. This functional polymorphism was shown to affect the ability to perform verbal episodic memory tasks and hippocampal function [4], to influence *BDNF* mRNA localization, putatively impairing dendritic targeting of *BDNF* transcript [30] and to alter the intracellular distribution and activity-dependent secretion of the BDNF protein [31].

MicroRNAs (miRNAs) are small non-coding RNAs of about 22-nucleotides in length regulating gene expression at post-transcriptional level. Once processed from longer stem-loop-like precursors they are guided to target mRNA sequences by base-pairing 3′UTR, resulting in the cleavage of target mRNAs or repression of their translation [32]. The seed region is the critical region for miRNA binding to the mRNA target site by Watson-Crick complementariness and consists of nucleotides 2–8 from the miRNA 5′ [33]. To date, miRNAs have been shown to be involved in many physiological processes, such as differentiation, proliferation, apoptosis and morphogenesis [34] and pathological events, i.e. cardiac hypertrophy [35], muscle dystrophy [36], hepatitis infection [37], diabetes [38], Parkinson's disease [39], haematological malignancies [40] and other types of cancer [41] and psychiatric and neurodevelopmental disorders [42]–[44].

Human *BDNF* expression is controlled by complex mechanisms, indeed its transcription is regulated by multiple promoters driving the expression of different coding transcripts [45]. It has been recently observed that genes bearing multiple binding sites for transcription factors (TF) show higher probabilities to be targeted by miRNAs and to harbour more miRNA-binding sites on average [46]. This observation indicates that genes with higher cis-regulation complexity are more co-coordinately regulated by TFs at transcriptional level and by miRNAs at post-transcriptional level [46]. This has been partly demonstrated by *in silico* analysis of human *BDNF* 3′UTR sequence using bioinformatics tools predicting the presence of several putative miRNA target sites. To date some of these sites have been experimentally validated (miR-1/206 [47]; miR-30a, miR-30a-5p and miR-195 [48]; miR-124 and let-7d [49]; miR-15a [50]; miR-210 [51]).

Recent studies on human miRNA target sequences suggested that the nucleotide variants mapping in these regions could alter miRNA directed translation silencing likely contributing to diversity of human phenotypes [52], [53]. Such mechanism was described in two patients affected by Tourette syndrome harbouring a 3′UTR rare sequence variant in the *SLITRK1* gene reinforcing a target site for miR-189 [54]. Since then, several papers demonstrated the effects of miRNA binding sites variations in asthma, hypertension and aggressive behaviour [55]–[57].

In the present work we demonstrated through *in silico* and experimental approaches that *BDNF* expression is regulated by a group of miRNAs. We also investigated whether common allelic variants in *BDNF* sequence may influence miRNA targeting and thus participate to *BDNF* expression modulation. We identified two common polymorphisms (rs11030100 and rs11030099) mapping to the 3′UTR of the longest *BDNF* mRNA isoform and demonstrated that they modulate the interaction with two different miRNAs. Finally, we considered the rs6265 coding SNP, which was previously associated to various neuropsychiatric diseases and assessed the haplotype containing this variant and the two 3′ UTR polymorphic sites within a schizophrenic and a control sample.

## Results

### Bioinformatics analysis

Bioinformatics approaches have been applied to identify miRNAs potentially binding to human *BDNF* 3′UTR and to assess the presence of sequence variations in miRNA binding sites. From the analysis of PolymiRTS Database [58] three polymorphic sites were identified (rs11030100 C>A, rs11030099 G>T and rs7124665 C>A, dbSNP build 130) (Table 1). The first two SNPs were predicted to affect the same hypothetical seed region of miR-26a (encoded by two different genomic loci miR-26a1 and miR-26a2) and miR-26b. The third polymorphism (rs7124665) was predicted to create a new binding site for miR-374. From the Predicted Targets component of miRecords miR-26a and -26b are predicted to target *BDNF* by at least 3 and 2 different programs respectively, miR-374 at least by 2 programs (Table 2).

**Table 1:** SNPs that modify miRNA binding sites according to PolymiRTS Database.

Location	SNP ID	Ancestral Allele	Allele	miR ID	(a) Support	miRSite	(b) Func Class
2884	rs11030100	C	C	miR-26a miR-26b	1	ccaagACTTGAAg	N
			A				
2887	rs11030099	G	T				
			G	miR-26a miR-26b	1	agACTTGAAggtg	N
3383	rs7124665	C	C				
			A	miR-374	0	TATTATAtttgta	C

PolymiRTS Database uses the criteria of TargetScan for miRNA binding sites prediction.

(a) Support column indicates occurrence of the miRNA site in other vertebrate genomes in addition to the query genome.

(b) Function Class specifies if the derived allele either disrupts a non conserved miRNA site (N) or creates a new miRNA site (C).

**Table 2:** Analysis output with miRecords, that integrates predicted miRNA targets produced by 11 miRNA target prediction programs.

miRNA ID	miRanda	PITA	RNAhybrid
hsa-miR-26a	√	√	√
hsa-miR-26b	√	√	
hsa-miR-374a	√	√	
hsa-miR-374b		√	

Here are reported programs that predict *BDNF* 3′UTR miRna binding sites for miRNAs identified with PolymiRTS. Other programs are: DIANA-microT, MicroInspector, MirTarget2, miTarget, NBmiRTar, PicTar, TargetScan and RNA22.

### Genotype analysis of *BDNF* 3′UTR polymorphisms

**Figure 1:**
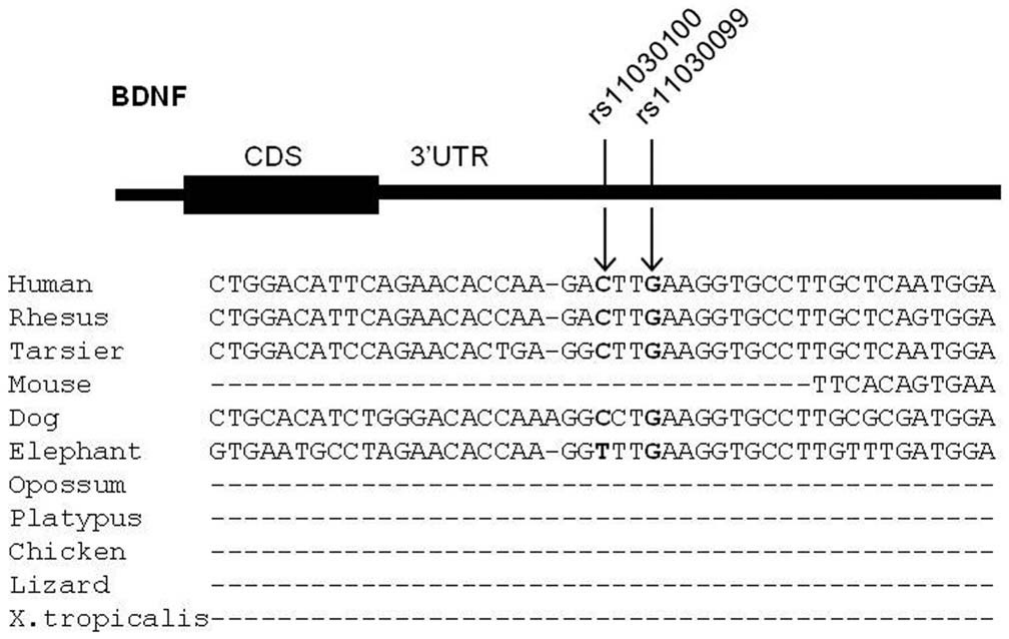
Multiple genome alignment of *BDNF* 3′UTR in the region of polymorphic sites rs11030100 and rs11030099 (in bold letters). Sequence analysis shows that sites are highly conserved among primate genomes.

A group of 176 Caucasian control subjects was collected in order to estimate allelic frequencies at the polymorphic sites. rs7124665 resulted not to be variable, while rs11030100 and rs11030099, mapping two nucleotides apart one from the other, were polymorphic (Table 3). Genotypic distribution was in Hardy-Weinberg equilibrium for both variants (chi^2^ = 1.99, *p* = 0.16, df = 1). The ancestral allele of both SNPs, was determined by human versus chimpanzee (*Pan troglodytes*) genome alignment and it was always observed in the same haplotype suggesting that the two variants could have arisen in a single mutational event. MiRNA binding site sequences containing the two polymorphic sites have been analyzed through multiple genome alignment and showed high sequence conservation among primate genomes (Fig. 1).

**Table 3:** Allele and genotype frequencies of *BDNF* polymorphisms rs11030100-rs11030099 in control subjects and schizophrenic patients.

	Genotypes			Alleles		N
*BDNF* rs11030100-rs11030099	C/C-G/G	C/A-G/T	A/A-T/T	C–G	A–T	
**Control subjects**	0.57	0.39	0.04	0.77	0.23	176
**Schizophrenic patients**	0.68	0.26	0.06	0.81	0.19	70

### MiR-26a and -26b interact with human *BDNF* 3′UTR

**Figure 2:**
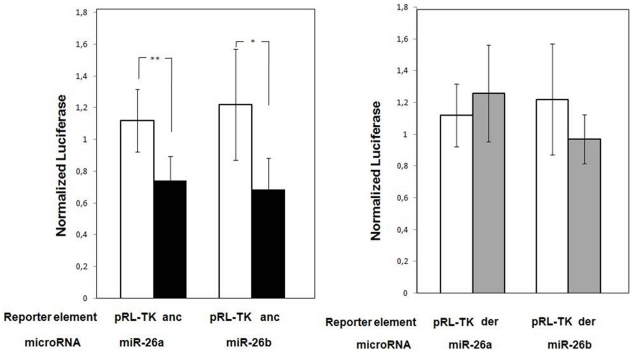
Luciferase assays for validation of miR-26a and -26b binding to *BDNF* 3′UTR. HeLa cells were independently transfected with control plasmid (pRL-TK) or each of the two reporter plasmid (pluc-BDNF C–G, anc, and pluc-BDNF A–T, der) with either miR-26a or miR-26b. Data are presented as the normalized activity of different reporter genes. Introduction of exogenous miR-26a and miR-26b represses reporter activity of pluc-BDNF C–G but has no effect on pluc-BDNF A–T. Data represent the mean of five independent experiments +SD (*p*<0.05).

We focused the experiments on miR-26a and -26b since *in silico* analysis predicted these miRNAs as targeting *BDNF* through base pairing to 3′UTR sites that resulted to be polymorphic from genotype analysis (rs11030100 and rs11030099). In order to functionally validate the computational data, a dual-luciferase assay was performed in HeLa cells. The activity of the reporter plasmid with ancestral alleles at polymorphic sites (pluc-BDNF C-G) in the presence of each of the two miRNAs was significantly lower than those of cells transfected with miR-control or unmodified pRL-TK vector, indicating that *BDNF* was specifically down-regulated by miR-26a and miR-26b miRNA mimics (33% and 43% respectively, Fig. 2).

From these results we could conclude that human *BDNF* is targeted by miR-26a and miR-26b which bind to the same seed sequence.

### *BDNF* allelic variants decrease miR-26s binding ability

**Figure 3:**
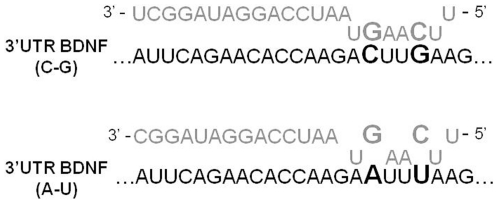
Schematic representation of base pairing between miR-26a sequence and *BDNF* 3′UTR ancestral (C–G) and derivative (A–T) alleles of rs11030100 and rs11030099 polymorphic sites. MiR-26b has the same seed binding sequence.

Sequence analysis demonstrated that the derivative alleles of rs11030100 (A) and rs11030099 (T) were contemporaneously present for both SNPs. Since those variants created two potential mismatches between *BDNF* 3′UTR seed sequence and miR-26a and 26b (Fig. 3) we hypothesized that they may impair miR-26s targeting. To validate this hypothesis, we tested the interaction between mutant *BDNF* transcripts and both miR-26a and -26b. In agreement with our assumption, when the reporter constructs were cotransfected with either miR-26a or miR-26b, no modification of the luciferase signal of the pRL-TK vector carrying the derivative alleles (pluc-BDNF A-T) could be observed (Fig. 2), as obtained with miR-control or unmodified pRL-TK. We could conclude that allelic variants at rs11030100 and rs11030099 sites abrogate miR-26s mediated translation suppression.

### Genotype analysis of *BDNF* miR26-a and –b binding site in a schizophrenic sample

In order to analyze the allelic distribution of the identified polymorphisms in a psychiatric disease sample, a group of 70 schizophrenic patients was collected and genotyped for the fragments containing rs11030100, rs11030099 and rs7124665. As for the control group (see Results section, “*Genotype analysis of BDNF 3′UTR polymorphisms”* paragraph), the latter SNP resulted not to be polymorphic, while rs11030100 and rs11030099 were polymorphic. The ancestral allele of both SNPs was always observed in the same haplotype combination (see Table 3 for allele and genotype frequencies) and the genotypic distribution for each polymorphism was in Hardy-Weinberg equilibrium (chi^2^ = 1.57, *p* = 0.21, df = 1). No significant differences in the allelic distribution were observed between case and control samples for each SNP.

### Genotype analysis of rs6265 coding SNP

rs6265 SNP was genotyped both in case and control subjects. Met allele frequency (Table 4) was consistent with reported allele frequency in European ancestry group (dbSNP 130). The genotypic distribution of rs6265 for both groups of cases and controls were in Hardy-Weinberg equilibrium (chi^2^ = 3.38, *p* = 0.07, df = 1 and chi^2^ = 0.40, *p* = 0.53, df = 1 respectively). No significant differences in the allelic distribution of rs6265 and rs11030100-rs11030099 in the schizophrenic and control samples was evidenced.

**Table 4:** Allele and genotype frequencies of *BDNF* polymorphism rs6265 (Val66Met) in control subjects and schizophrenic patients.

	Genotypes			Alleles		N
*BDNF* Val66Met (rs6265)	Val/Val	Val/Met	Met/Met	Val	Met	
**Control subjects**	0.63	0.34	0.03	0.80	0.20	176
**Schizophrenic patients**	0.73	0.21	0.06	0.84	0.16	70

### Pairwise linkage disequilibrium between *BDNF* polymorphisms and haplotype association analysis

In order to assess the level of linkage disequilibrium (LD) between rs6265 and rs11030100 and rs11030099 we studied the *BDNF* genomic region. We assessed the haplotype structure for 43 control subjects (/139) and 15 patients (/70) samples who resulted to be heterozygous for the coding SNP rs6265 and for the 3′UTR SNPs (rs11030100 and rs11030099) through amplification and cloning of a fragment comprising the polymorphic sites. The haplotype structure and the LD extent in both samples have been computationally predicted and experimentally assessed through double restriction enzyme analysis. A nearly complete LD was found between the three SNPs in both case and control group. The extent of LD between the polymorphic sites was determined by calculating the D′ statistics. LD (D′) calculated and estimated (in parenthesis) in control subjects and schizophrenic samples was 0.89 (0.91) and 0.89 (1) respectively.

Four haplotypes consisting of alleles for the *BDNF* SNPs were observed. The most represented haplotype was “Val-C-G” (respectively rs6265, rs11030100, rs11030099) showing a frequency of 75% in control and 80% schizophrenic sample. The second most frequent haplotype was “Met-A-T” which accounted for 19% in control and 15% in schizophrenic subjects. Other haplotypes were less frequent (Val-A-T 4% in both groups and Met-C-G 2% and 1%). The comparison of haplotype frequencies between cases and controls revealed no significant differences.

## Discussion

The regulation of mRNA stability and translation through miRNAs represents a mechanism directing and influencing gene expression widely described in mammals [34]. DNA sequence variations at miRNA binding sites could affect the base-pairing process between a miRNA and its target, hence making the miRNA-mediated gene repression ineffective and putatively leading to phenotypic effects [59]–[60]. Indeed, it has been recently suggested that such events underlie the onset of cancer [61] and various other pathological conditions [55]–[57].

Using different bioinformatic tools we identified a group of miRNAs, namely miR-26a-1, miR-26a-2, miR-26-b and miR-374, supposedly regulating *BDNF* translation by binding to three annotated polymorphic sites. MiR-26a1, -26a2 and -26b bind to a *BDNF* 3′UTR sites whose sequence is not conserved among mammals. This may explain the small number of programs predicting this putative binding site since many algorithms mainly depend on conservation over a large range of species, thus target prediction results biased against newly evolved miRNA target sites. Interestingly, narrowing the genome comparison analysis only to higher primates, miR-26s seed binding sequence results to be highly conserved suggesting that this regulatory circuitry could be recently acquired in primate evolution.

To date *BDNF* 3′UTR structure has been poorly characterized and most studies focused on 5′UTR and coding sequence [62]. Several studies have focused on rat *BDNF* gene structure which can be alternatively polyadenylated directing the synthesis of at least two transcripts differing in 3′UTR size [63]–[64] and exhibiting different properties such as the turnover rate at activated synapses [65]. More recently it has been demonstrated in a murine model that these two isoforms show a diverse neuronal localization [66] and that the distinct 3′UTRs differentially regulate *BDNF* translation in response to neuronal activity changes [67]. Multiple polyadenylation and highly conserved sequences have been also reported in human genome [68] leading to the hypothesis that the same regulatory mechanism may exist in humans as well. MiR-26s binding site is exclusively present in the long *BDNF* mRNA isoform suggesting that miRNAs mediated regulation could account for the differential properties of *BDNF* mRNA isoforms such as stability and localization.

MiR-26a1, -26a2 and -26b belong to a miRNAs family showing a highly conserved sequence and sharing the same seed sequence. They are coded by different genome loci which map in the intragenic regions of paralog genes (*CTDSPL*, *CTDSP2*, *CTDSP1*). As we demonstrate that miR-26s binding site is polymorphic (rs11030100 and rs11030099), a regulatory effect could be exerted by the affinity rank between miRNA and its binding site. In particular, as the target for both miR-26a and -26b is exclusively the rs11030100 and rs11030099 ancestral allele only the *BDNF* mRNA carrying the ancestral genotype would be regulated by these miRNAs. Noteworthy, miR-26s are differentially expressed in tissues and are highly expressed in brain where they play a crucial role in neural cells specification [69], [70]. These observations taken together with the expression data available in public databases lead to hypothesize the existence of an overlapping expression pattern of miR-26s and *BDNF* in brain so that the 3′UTR SNPs abrogating miRNA regulation would hold a functional role only in those areas expressing the cognate miRNAs.

We further extended the analysis to rs6265 SNP which maps in the coding sequence of pro-BDNF and which is the best characterized *BDNF* functional variant to date. rs6265 is associated with modulation of intracellular trafficking and packaging of pro-BDNF, that acts as a dimer, and consequently with the secretion of the activity-dependent mature peptide [4], [31]. It has been demonstrated that this SNP has an effect also on *BDNF* mRNA localization, putatively impairing dendritic targeting of *BDNF* transcript [30]. Interestingly, nearly complete LD between rs6265 and SNPs rs1130100 and rs11030099 was found both in case and control samples allowing to hypothesize that miR-26s mediated regulation could extend to rs6265 leading to an allelic imbalance potentially modulating its functional effect on both mRNA and protein. In particular, it could be assumed that the most represented haplotype carrying the ancestral alleles at each polymorphic site could contribute to regulate the expression of the more functional Val allele in modulation of intracellular trafficking leading to proper secretion of the activity-dependent mature peptide.

The involvement of rs6265 in the susceptibility to various psychiatric disorders and especially schizophrenia has been extensively investigated through linkage and association studies leading to conflicting results [25]–[29]. Since large allelic frequencies variations of the rs6265 SNP have been reported in population of different ethnic origins, it has been proposed that a yet unidentified causal variant could possibly lie within or nearby *BDNF*. On the other hand, literature data about miR-26a and miR-26b pointed out some evidences about the role of these miRNAs in brain functions, such as stress response [70], and in schizophrenia susceptibility [71]–[72]. In particular, the association between schizophrenia and sequence variants in miRNA genes was evaluated in a case-control study demonstrating that a polymorphism mapping to the genomic region adjacent to miR-26a-1 showed allelic association to schizophrenia [71]. Recently it has been reported that miR-26b was expressed at lower levels in the prefrontal cortex of schizophrenia versus comparison subjects [72]. Therefore, we studied the distribution of rs6269, rs11030100 and rs11030099 in a sample of schizophrenic subjects and compared the results with the normal control sample. No significant differences in the allelic and haplotypic distribution emerged from the comparison of schizophrenic and control subjects but this result may not be not conclusive because of the small sample size. It would be crucial to study the involvement of these variants in psychiatric disorders pathogenesis in large case-control samples.

Provided that *BDNF* structure and expression is extremely complex, as recently stated by several studies, detailed knowledge of novel regulatory sequences may contribute to understand cell-specific and activity-dependent regulation of *BDNF* expression offering new interesting information that could be related to physiologic and pathogenetic mechanisms in which *BDNF* is involved.

The present study demonstrated that miR-26a and miR-26b mediate *BDNF* expression regulation and lead to identity the first *BDNF* 3′UTR functional variants (rs1130100 and rs11030099) altering miRNAs-*BDNF* binding. These polymorphisms have been evaluated in a haplotypic context showing nearly complete LD with rs6265 coding SNP.

## Materials and Methods

### Bioinformatic analysis

Information about *BDNF* genomic region were obtained from the genome browser of the University of California, Santa Cruz (http://genome.ucsc.edu). *BDNF* gene and mRNA sequences were from RefSeq (http://www.ncbi.nlm.nih.gov/RefSeq) and GenBank (http://www.ncbi.nlm.nih.gov/Genbank) databases. PolymiRTS (Polymorphism in miRNA Target Site) database was interrogated (http://compbio.uthsc.edu/miRSNP) to identify sequence variations in putative miRNA binding sites. MiRecords (http://mirecords.biolead.org/) was used to predict binding sites for miRNAs on *BDNF* 3′UTR. Information about *BDNF* and miRNAs expression was obtained from public databases (http://www.genecards.org, http://biogps.org, http://mirnamap.mbc.nctu.edu.tw).

### Subjects

70 patients with schizophrenia (35 males; mean age ± SD, 28.45±6.32 years) and 176 healthy subjects (45 males; mean age ± SD, 32.88±9.74 years) were recruited for the present study at Department of Neuroscience, University of Rome Tor Vergata. All participants were unrelated, white Caucasians from Rome and surrounding urban areas. Diagnosis of schizophrenia was reached by consensus between two experienced psychiatrists by using DSM-IV-TR based Structured Clinical Interview (SCID). Healthy subjects underwent SCID to exclude any DSM-IV-TR Axis I disorder. None of the patients and controls had a history of significant drug or alcohol abuse, head trauma with loss of consciousness, or significant medical illness.

### Ethics statement

Informed written consent was obtained from all control subjects and patients prior to inclusion and the study was approved by the local research ethical committees where patients were recruited (University of Rome Tor Vergata). Psychiatrists who made the schizophrenia diagnosis provided advice regarding the patients' capacity to provide informed consent. The Ethic Committee discussed this point and approved the methodological procedure to obtain the informed written consent from all subjects included in the study.

### Genotype analysis

Genomic DNA was isolated from peripheral nuclear blood cells according to standard procedures. All subjects included in the present study were genotyped for rs11030100, rs11030099, rs7124665 and rs6265 *BDNF* polymorphisms through Sanger sequencing. Primer oligonucleotides used for amplification through PCR were respectively: 5′-GAGGTGGCTCTGGAATGACATG-3′ and 5′-CAGCAGATATTCCAAGCATTCC-3′; 5′-GCAATTGCTGCATCTTAGTAGG-3′ and 5′-GCCTCCCAGGCTTTCAAATAAG-3′; 5′–GGTGCAGCTGGAGTTTATCAC-3′ and 5′-GGTCTCGTAGAAGTATTGCTTC-3′. Amplification reaction was performed in a total volume of 25 µl containing: 200 ng of DNA template, 200 µM of dNTPs (NEB, Ipswich, MA), 0.3 µM of each primer, 1.5 mM MgCl_2_, PCR buffer 1× and 1.5 U of AmpliTaqGold polymerase (Applied Biosystems, Foster City, CA). An initial denaturation step at 94°C for 11 min was followed by 30 cycles of amplification at 94°C for 30 sec, 59°C for 30 sec and 72°C for 30 sec. PCR products were sequenced using Big Dye terminator cycle sequencing kit v3.1 (Applied Biosystems) according to manufacturer's recommended protocols. The sequences were analyzed on an automatic sequencer (3100 Applied Biosystems).

### Cell Culture

HeLa cells (ATCC CCL-2) were grown in Dulbecco's modified Eagle's medium (DMEM, Invitrogen), supplemented with 200 U/ml penicillin, 200 µg/ml streptomycin and 10% heat inactivated foetal bovine serum (FBS) at 37°C in 95% humidifier air and 5% CO2.

### Luciferase activity assay

3′UTR *BDNF* reference and variant sequences were amplified from human genomic DNA. The reaction was performed in a total volume of 25 µl containing: 100 ng of DNA template, 200 µM of dNTPs (NEB), 1.2 µM of each primer (5′-GTCTAGAGTCTTAAAGCAAGGAACACACG;5′-GTCTAGACTTCTTGTGTATGTACATTGACC), PCR Buffer3 1× and 3.75 U of Expand High Fidelity Enzyme mix (Roche). An initial denaturation step at 94°C for 2 min was followed by 30 cycles (15 sec at 94°C, 30 sec at 60°C, 1 min at 72°C) and then by a final extension step at 72°C for 7 min. Fragments were subcloned into the XbaI site in the 3′UTR of Renilla luciferase of pRL-TK reporter vector containing the promoter and the enhancer to express Renilla luciferase in cotransfected cells. Reporter plasmids (150 ng of pRL-TK reporter vector, 50 ng of reporter firefly construct - pGL3 - to standardize for luciferase assay and 100 nM of miRNA mimics, Dharmacon, Lafayette, CO) were transfected into HeLa cells (70% confluence) using Lipofectamine 2000 (Invitrogen) according to the manufacturer's instructions. The transfection was performed in Opti-MEM (Invitrogen), which was removed after 4 h and replaced with Dulbecco's modified Eagle's medium containing 10% fetal bovine serum. After 24 h cells were lysated and reporter activity was determined using Dual-luciferase report assay system (Promega, Madison, WI) according to the manufacture's protocols. Renilla luciferase activity was normalized to firefly luciferase activity. Values of luciferase activity were compared using Student's t test.

### Haplotype analysis

The haplotype structure of subjects heterozygotes for rs6265 and rs11030100/rs11030099 (43 control subjects and 15 patients) was realized through amplification and cloning of a 3030 bp fragment containing the three polymorphic sites. PCR was performed in a total volume of 50 µl containing: 250 ng of DNA template, 250 µM of dNTPs (NEB), 1 µM of each primer (5′-GGGAAACACTGCATGTCTCTG-3′; 5′-ATGCTGGTCCAAGTGGTGATC-3′), PCR Buffer3 1× and 1,875 U of Expand Long Template PCR system (Roche, Indianapolis, IN). An initial denaturation step at 94°C for 3 min was followed by 30 cycles of amplification at 94°C for 30 sec, 65°C for 40 sec and at 68°C for 4 min. The haplotype structure and the LD extent in both samples were computationally predicted and experimentally assessed through double restriction enzyme analysis. Fragments were subcloned in pCR2.1 vector (TA Cloning Kit, Invitrogen, Carlsbad, CA) according to the manufacturer's recommended protocols and subject to enzymatic digestion using PmlI and of ApoI to detect alleles at rs6265 and rs11030100/rs11030099 sites, respectively.

### Statistical analysis

Hardy-Weinberg equilibrium of the genotypic distributions was examined using the chi^2^ test for goodness of fit. The differences in genotype and allele frequencies of the observed variants between cases and controls were evaluated by the chi^2^ test. Statistical significance was defined as *p*<0.05. Statistical analysis was conducted using SHEsis software (http://analysis.bio-x.cn [74]). LD values between alleles of rs6265 and rs11030100/rs11030099 were obtained using the expectation-maximum method (Arlequin v. 3.11 [73]).
